# Trends and Disparities in Access to Buprenorphine Treatment Following an Opioid-Related Emergency Department Visit Among an Insured Cohort, 2014-2020

**DOI:** 10.1001/jamanetworkopen.2022.15287

**Published:** 2022-06-03

**Authors:** Maria A. Stevens, Jennifer Tsai, Samuel T. Savitz, Bidisha Nath, Edward R. Melnick, Gail D’Onofrio, Molly Moore Jeffery

**Affiliations:** 1 Department of Health Care Policy Research, Mayo Clinic, Rochester, Minnesota; 2 Department of Health Policy and Management, University of North Carolina at Chapel Hill; 3 OptumLabs, Eden Prairie, Minnesota; 4 Department of Emergency Medicine, Yale School of Medicine, New Haven, Connecticut; 5 Department of Emergency Medicine, Mayo Clinic, Rochester, Minnesota

## Abstract

This cross-sectional study examines trends in access to buprenorphine treatment following an opioid-related emergency department (ED) visit among adults with commercial or Medicare Advantage health insurance between 2014 and 2020.

## Introduction

Emergency department (ED)–initiated buprenorphine is a first-line treatment^1^ for opioid use disorder (OUD) that is cost-effective and saves lives but remains underused^2,3^ amid access disparities.^4^ Prior cross-sectional analyses^5,6^ evaluating trends in ED buprenorphine using national data did not assess trends in disparities. We describe recent national trends in access to buprenorphine and disparities in access after an opioid-related ED visit in an insured cohort.

## Methods

This cross-sectional study included people with commercial or Medicare Advantage health insurance between 2014 and 2020 from the OptumLabs Data Warehouse (OLDW). OLDW contains deidentified longitudinal administrative claims and enrollment data. New buprenorphine fills (excluding formulations for pain) within 7 days following an opioid-related ED visit were reported per 10 000 opioid-related ED visits, identified using a validated phenotype (eAppendix in the Supplement). We used *Current Procedural Terminology* and revenue codes from professional and facility claims to identify ED visits and diagnosis codes for opioid use, abuse, dependence, and poisoning to identify opioid-related ED visits (eAppendix in the Supplement). ED visits resulting in a hospital admission were excluded. Patient demographics including sex, age, and race and ethnicity were ascertained from OLDW; race and ethnicity were assessed as factors associated with disparities in access to buprenorphine treatment. This study was conducted using deidentified data and, as such, did not require institutional review board approval or informed patient consent, in accordance with 45 CFR §46. This study followed the Strengthening the Reporting of Observational Studies in Epidemiology (STROBE) reporting guideline.

Buprenorphine fill rates were plotted for 2014 to 2015, 2016 to 2017, 2018 to 2019, and 2020. When reporting on subpopulations, rates were standardized to the number of opioid-related ED visits for each subpopulation. Percentage changes were calculated between 2014 to 2015 and 2018 to 2019 because of COVID-19–related interruptions in care that may affect continuity of trends through 2020. Associations between buprenorphine fills and visit characteristics were assessed using Pearson χ^2^ tests. Two-sided *P *<* *.05 was considered significant. Analyses were conducted using Stata statistical software version 17.0 (StataCorp) from October 2021 to March 2022. Additional detail on study methods is available in the eAppendix in the Supplement.

## Results

We identified 1813 buprenorphine fills from 72 055 opioid-related outpatient ED visits. From 2014 and 2015 to 2018 and 2019, fills per 10 000 opioid-related ED visits increased from 197 to 301 (53.3% [95% CI, 31.0%-79.4%]). Fills per 10 000 opioid-related ED visits increased by a significant amount for all populations except for non-Hispanic Black and Hispanic populations (Figure). Across pre–COVID-19 pandemic study years and through 2020, fills were generally lower for female (vs male) individuals, people aged at least 41 years (vs aged 18-25 years and 26-40 years), and non-Hispanic Black and Hispanic (vs non-Hispanic White) populations (Table and Figure).

**Figure.**
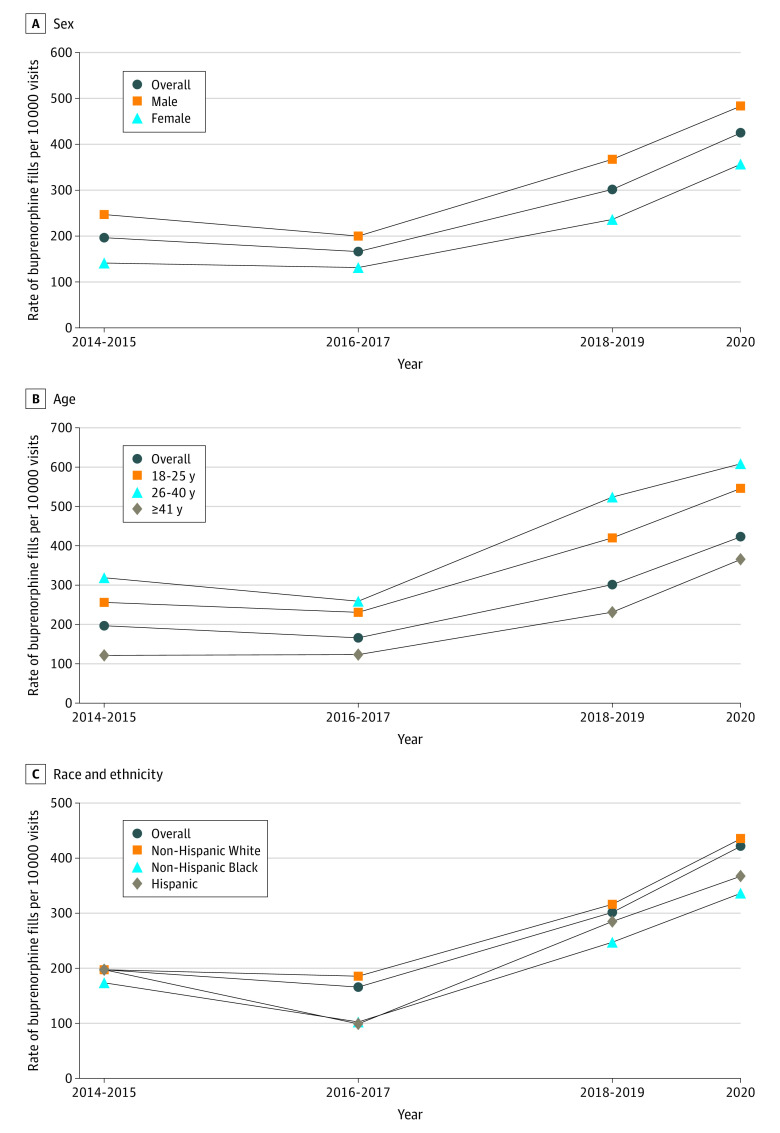
Buprenorphine Fills Per 10 000 Opioid-Related Emergency Department Visits and Percentage Changes From 2014-2015 to 2018-2019 by Sex, Age, and Race and Ethnicity The overall percentage change from 2014-2015 to 2018-2019 was 53.3% (95% CI, 31.0% to 79.4%). In panel A, the percentage change was 48.6% (95% CI, 22.1% to 81.0%) for men and 67.7% (95% CI, 29.3% to 117.7%) for women. In panel B, the percentage change was 64.0% (95% CI, 19.0% to 126.1%) for patients aged 18 to 25 years, 64.6% (95% CI, 25.7% to 115.5%) for patients aged 26 to 40 years, and 90.3% (95% CI, 47.2% to 146.1%) for patients aged 41 years and older. In panel C, the percentage change was 60.5% (95% CI, 34.1% to 92.2%) for White patients, 42.4% (95% CI, −11.3% to 128.6%) for Black patients, and 44.0% (95% CI, −18.5% to 154.6%).

**Table.** Opioid-Related ED Visits by Receipt of Buprenorphine Fills and Visit Characteristics, 2014-2020

Characteristics	Visits, No. (%)		*P* value
	No buprenorphine (n = 70 242 [97.5%])	Buprenorphine (n = 1813 [2.5%])^a^	
Sex			
Male	35 259 (97.0)	1104 (3.0)	<.001
Female	34 983 (98.0)	709 (2.0)	
Age, y			
18-25	9568 (96.9)	307 (3.1)	<.001
26-40	13 376 (96.1)	547 (3.9)	
≥41	47 298 (98.0)	959 (2.0)	
Race and ethnicity			
Asian or unknown^b^	4108 (96.8)	137 (3.2)	<.001
Hispanic	6390 (97.9)	138 (2.1)	
Non-Hispanic			
Black	10 406 (98.0)	208 (2.0)	
White	49 338 (97.4)	1330 (2.6)	
Year of opioid-related ED visit			
2014	5511 (97.5)	144 (2.6)	<.001
2015	7258 (98.5)	112 (1.5)	
2016	12 569 (98.7)	161 (1.3)	
2017	12 758 (98.0)	266 (2.0)	
2018	11 077 (97.5)	279 (2.5)	
2019	10 966 (96.4)	406 (3.6)	
2020	10 103 (95.8)	445 (4.2)	

Abbreviation: ED, emergency department.

^a^ Defined as receipt of buprenorphine within 7 days after an ED visit.

^b^ Listed as unknown in data source.

## Discussion

Our findings indicate that timely buprenorphine fills following an opioid-related ED visit increased but with disparities across sex, age, and race and ethnicity, with continued disparities observed for non-Hispanic Black and Hispanic (vs non-Hispanic White) populations. In this cohort of people with health insurance who were seen in the ED with an opioid-related diagnosis, people with socioeconomic advantages—being male, younger, or non-Hispanic White—were more likely to receive this life-saving treatment.

These findings are not surprising given that patient-level and system-level barriers for accessing buprenorphine^2^ often disproportionately affect minoritized racial and ethnic populations. These barriers include—but are not limited to—systemic racism, mistrust of clinicians and the health care system, insufficient supply of waivered prescribers, cumbersome reimbursement practices (eg, prior authorizations), fragmented care, social factors, and addiction and mental health–related stigma.

Study limitations included an inability to observe prescriptions and health services not submitted to the insurance plan, such as from methadone clinics, or whether buprenorphine fills were directly prescribed from the ED. This study describes associations rather than causation; results may not be generalizable beyond the commercial and Medicare Advantage population.

ED-initiated buprenorphine holds promise for helping to address the OUD treatment gap. However, clinical and policy remedies are needed to continue to increase buprenorphine treatment for OUD in EDs that target key disparities.

## Notes

Supplement. **eAppendix.** Supplemental Methods
