# Supplementary material for: Trends and Disparities in Access to Buprenorphine Treatment Following an Opioid-Related Emergency Department Visit Among an Insured Cohort, 2014-2020
Source: JAMA Netw Open. 2022 Jun 3;5(6):e2215287. doi: 10.1001/jamanetworkopen.2022.15287 (PMC9166266; doi:10.1001/jamanetworkopen.2022.15287)
Supplement: Supplement. — eAppendix. Supplemental Methods [file jamanetwopen-e2215287-s001.pdf]

Supplemental Online Content

Stevens MA, Tsai J, Savitz ST, et al. Trends and disparities in access to buprenorphine treatment following an opioid-related emergency department visit among an insured cohort, 2014-2020. *JAMA Netw Open.* 2022;5(6):e2215287. doi:10.1001/jamanetworkopen.2022.15287

eAppendix. Supplemental Methods

This supplemental material has been provided by the authors to give readers additional information about their work.

© 2022 Stevens MA et al. *JAMA Network Open.*

eAppendix. Supplemental Methods

Data

This study used de-identified administrative claims data from the OptumLabs Data Warehouse (OLDW),[1,2] which includes medical and pharmacy claims, laboratory results, and enrollment records for commercial and Medicare Advantage (MA) enrollees. The database contains longitudinal health information on enrollees and patients, representing a diverse mixture of ages, ethnicities and geographical regions across the United States. Since this study involved analysis of pre-existing, de-identified data, it was exempt from Institutional Review Board approval.

Sample

The sample included opioid-related ED visits from enrollees during the study period with commercial or Medicare Advantage health insurance. Codes used to identify opioid-related ED visits are based on a validated phenotype[3] and displayed in Table S1.

Eligibility

Inclusion criteria included having: 1) at least one medical claim for an opioid-related ED visit (excluding hospital admissions) during a period of insurance enrollment; 2) commercial or MA medical and pharmacy coverage; 3) at least 14 days of continuous enrollment prior to the opioid-related ED visit and at least 7 days of continuous enrollment after the opioid-related ED visit; 4) no MOUD fills or injection procedures within 14 days prior to the opioid-related ED visit; 5) aged 18 or older at the time of the opioid-related ED visit.

The 14-day period used to assess whether the person was currently in an episode of MOUD use was empirically identified by analyzing observed gaps between the end date of a buprenorphine fill and the next fill date. We identified all MOUD buprenorphine fills for individual enrollees. We calculated the date on which each fill was estimated to end by adding the days' supply to the fill date from the pharmacy claim. The days' supply on the claim is estimated by the pharmacist filling the prescription based on the prescriber instructions. We then looked for the next MOUD buprenorphine fill for each enrollee and identified whether there was a gap between the end of the previous fill and the fill date of the new fill. For cases where there was a gap, we calculated length of the gap, ranging from 0 days (e.g., a fill runs out on April 1 and the next fill date is April 2; the prior fill ran out before the next fill date, but there was no gap in days of use) to 2429 days. For cases where another MOUD buprenorphine fill occurred, the median gap was 1 day (25th percentile: 1 day, 75th percentile: 4 days). 10.73% of people had a gap of more than 14 days.

Visits were also excluded when the enrollee had methadone or naltrexone treatment within 14 days of the opioid-related ED visit, however, these treatments were rarely observed.

Measures

Buprenorphine Prescription Fills Following an Opioid-Related ED Visit

The outcome was seven-day buprenorphine fill rates per 10,000 opioid-related ED visits. ED visits included in the rates are specific to the demographic group involved. For example, buprenorphine fills for female patients are reported per 10,000 opioid-related ED visits by female patients. This outcome was based on whether there was a claim for an initial buprenorphine

© 2022 Stevens MA et al. *JAMA Network Open.*

prescription fill that occurred within seven days of an eligible opioid-related ED visit. A seven-day time window was chosen to capture a new buprenorphine fill that was prescribed either directly in the ED or related to the ED visit as part of a warm handoff (e.g., to community-based addiction services). Formulations for pain management were excluded. (Table S2)

Sample Characteristics

Characteristics including sex (male, female), age (18-25, 26-40, ≥41), and race/ethnicity (non-Hispanic White (White), non-Hispanic Black (Black), Hispanic, Asian/Unknown) were ascertained from the OLDW. Sex and age were retrieved from OLDW enrollment files. Race/ethnicity categories (White, Black, Asian, Hispanic, unknown) were provided by OLDW. Due to small cell sizes, Asian and unknown were collapsed into one Asian/Unknown category.

Data analysis

Poisson regression was used to calculate percent changes, clustering at the patient-level to account for repeated visits.

Table 1. Identification of Opioid-Related Emergency Department Visits

**Emergency Department Visit**	
CPT	99281, 99282, 99283, 99284, 99285, 99291
Revenue	0450, 0451, 0452, 0453, 0454, 0455, 0456, 0457, 0458, 0459, 0981
**Opioid-Related Visit**	
Opioid Poisoning	
ICD-9/<br>ICD-10	T40.0 T40.1 T40.2 T40.3 T40.4; 965.01, E850.0; 965.00, 965.02, 965.09, E.850.1, E.850.2;<br>T40.1X (T401X1*, T401X2*, T401X3*, T401X4*);<br>T40.0X (T400X1*, T400X2*, T400X3*,T400X4*);<br>T40.2X (T402X1*, T402X2*, T402X3*, T402X4*);<br>T40.3X (T403X1*, T403X2*, T403X3*, T403X4*);<br>T40.4X (T404X1*, T404X2*, T404X3*, T404X4*)
Opioid Use, Abuse, and Dependence	
ICD-9/ICD-10	304.00-304.03, 305.50-305.53, 304.70-304.73, 965.00-965.02, 965.09, E85.00-E85.02, E93.50-E93.51; F1190, F11920-F11922, F11929, F1193, F1194, F11950-F11951, F11959, F11981, F11982, F11988, F1199; F1110, F11120-F11122, F1129, F1114, F11150-F11151, F11159, F11181, F11182, F11188, F1119; F1120, F11220-F11222, F11229, F1123, F1124, F11250, F11251, F11259, F11281, F11282, F11288, F1129

Table 2. Medications and Dosage Criteria Used to Exclude Buprenorphine Prescribed for Pain Management or Other Usage

Variable	Exclusion
Brand Name	Butrans, Belbuca, Buprenex
Dosage Form Description	Patch, Cartridge, Syringe, Powder, Vial
Drug Strength	< 1 mg or units listed as 'mcg'

© 2022 Stevens MA et al. *JAMA Network Open.*

Figure. Eligibility Flow Diagram

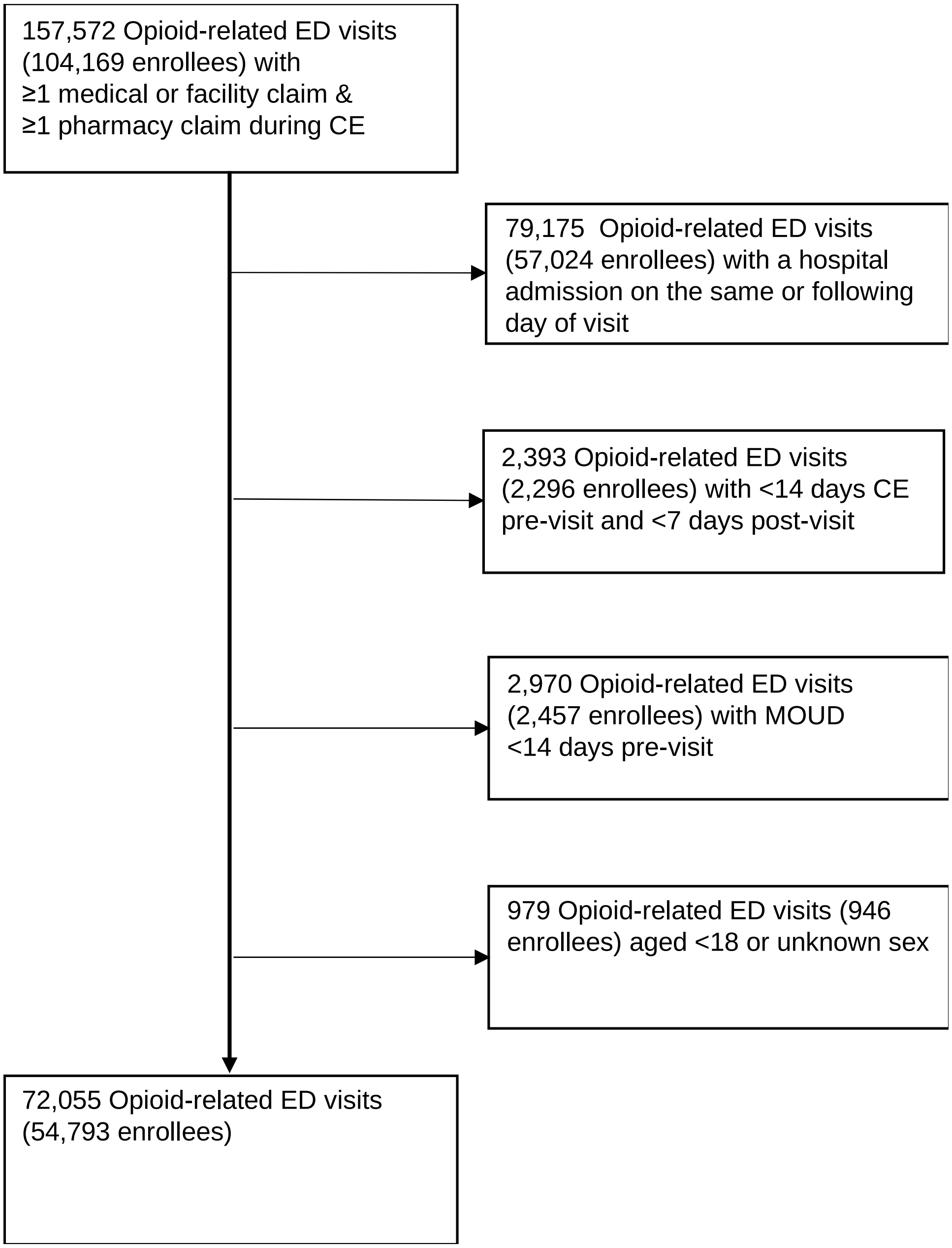

Note. ED=Emergency Department; CE=Continuous Enrollment; MOUD=Medication for Opioid Use Disorder

© 2022 Stevens MA et al. *JAMA Network Open.*

References

1. OptumLabs. *OptumLabs and OptumLabs Data Warehouse (OLDW) Descriptions and Citation.* Eden Prairie, MN: n.p., May 2019. PDF. Reproduced with permission from OptumLabs.

2. Wallace PJ, Shah ND, Dennen T, Bleicher PA, Crown WH. Optum Labs: building a novel node in the Learning health care system. Health Aff (Millwood). 2014 Jul;33(7):1187-94. doi: 10.1377/hlthaff.2014.0038.
Erratum in: Health Aff (Millwood). 2014 Sep;33(9):1703. Bleicher, Paul D [corrected to Bleicher, Paul A].PMID: 25006145.

3. Chartash D, Paek H, Dziura JD, et al. Identifying Opioid Use Disorder in the Emergency Department: Multi-System Electronic Health Record-Based Computable Phenotype Derivation and Validation Study. JMIR Med Inform. 2019;7(4):e15794. Published 2019 Oct 31. doi:10.2196/15794.

© 2022 Stevens MA et al. *JAMA Network Open.*
